# Health literacy and its sociodemographic and health determinants

**DOI:** 10.1590/1980-220X-REEUSP-2025-0441en

**Published:** 2026-06-12

**Authors:** Cristiane Ribas, Christiane de Fátima Colet, Edgar Noé Morelos-Garcia, Olga Maria Martins de Sousa Valentim, Márcio Strassburger, Fernanda Dal Maso Camera, Carolina Rodrigues Rusch, Adriane Cristina Bernat Kolankiewicz

**Affiliations:** 1 Universidade Regional do Noroeste do Estado do Rio Grande do Sul, Programa de Pós-Graduação Strictu Sensu em Atenção Integral à Saúde, Ijuí, RS, Brazil.; 2 Universidade Autônoma de Tamaulipas, Tamaulipas, Mexico.; 3 Universidade de Lisboa, Escola Superior de Enfermagem de Lisboa, Centro de Investigação, Inovação e Desenvolvimento em Enfermagem de Lisboa, Lisboa, Portugal.; 4 Universidade do Porto, Escola Superior de Enfermagem, RISE-Health, Porto, Portugal.; 5 Universidade Regional do Noroeste do Estado do Rio Grande do Sul, Curso de Fisioterapia, Ijuí, RS, Brazil.; 6 Universidade Regional Integrada do Alto Uruguai e das Missões, Programa de Pós-Graduação Strictu Sensu em Atenção Integral à Saúde, Erechim, RS, Brazil.; 7 Universidade Regional do Noroeste do Estado do Rio Grande do Sul, Curso de Enfermagem, Ijuí, RS, Brazil.

**Keywords:** Emergency Medical Services, Health Literacy, Health Behavior, Population Characteristics.

## Abstract

**Objective:**

To assess health literacy and its sociodemographic and clinical associations among users of an emergency care unit.

**Method:**

A cross-sectional study was conducted in a 24-hour emergency care unit in southern Brazil, with a sample of 550 users (≥18 years). A sociodemographic and clinical questionnaire was applied, along with scales 1 to 5 of the Health Literacy Questionnaire. Descriptive and inferential analyses were performed (Student’s t-test and ANOVA; p ≤ 0.05).

**Results:**

The highest averages were found in “social support” (2.95 ± 0.60) and “information assessment” (2.71 ± 0.44), and the lowest in “sufficient information” (2.64 ± 0.49) and “professional understanding” (2.69 ± 0.58). Higher scores were associated with female sex (p = 0.021), age >40 years old (p ≤ 0.044), living together (p = 0.045; p = 0.010), cohabitation (p = 0.008), higher income (p = 0.006; p = 0.007), and higher own or parents’ education level (p ≤ 0.038). Reading habits were associated with all dimensions (p ≤ 0.005). Health problem (p ≤ 0.023), adherence to treatment (p < 0.05), recent service search (p ≤ 0.037), and seek for information from professionals (p = 0.001) also influenced the scores.

**Conclusion:**

Health literacy varied significantly according to sociodemographic and clinical characteristics among users.

## INTRODUCTION

The Brazilian Public Health System (*SUS*) is structured on three hierarchical bases, considering the levels of health care: Primary, medium complexity, and high complexity care. The coordination of these levels is essential for adequate and efficient assistance, aiming at comprehensive patient care^(1)^. As part of the *SUS* healthcare services, the 24-hour Emergency Care Unit (24-hour *UPA*) is a service with intermediate complexity between Primary Health Care (PHC) and the Hospital Network. The service aims to guarantee effective care for users suffering from acute clinical conditions, as well as in surgical and trauma situations^(2)^.

In this context, Health Literacy (HL) is essential so that the population can take care of their health, follow up on treatments, adhere to them, and make decisions that benefit their health, which can directly impact the health quality of individuals and the community^(3)^. In 2020, the *US Department of Health and Human Services* defined HL as “the degree to which individuals have the ability to find, understand, and use information and services to inform health-related decisions and actions for themselves and others”^(4)^.

Likewise, the World Health Organization broadened the definition by stating that HL is not only an individual attribute, but also a product of social, economic, and institutional conditions. Therefore, it is up to health services to create environments, materials, and communication practices that support understanding, decision-making, and the exercise of autonomy, highlighting the role of health organizations in developing the population*’*s HL^(5)^.

HL has multiple concepts and is related to and intertwined with the fields of health and education, aiming to improve the individual*’*s quality of life and autonomy. To assess the strengths and weaknesses of individuals*’* HL^(6)^, one of the tools used is the *Health Literacy Questionnaire* (HLQ)^(7)^. This is a multidimensional instrument, applicable in different cultures and countries, with the aim of assessing HL, as well as cognitive reading and numeracy skills^(7–8)^.

Studies on HL have been conducted in different healthcare and population contexts, including PHC users with chronic conditions such as hypertension and type II diabetes mellitus^(9)^, as well as individuals undergoing dialysis therapy^(10)^, people in the emergency room^(11)^, with coronary artery disease^(12)^, with hematological cancer^(13)^, health professionals^(14)^, home caregivers^(15)^, and people living with severe and persistent mental disorders^(16)^. In general, these investigations show consistent associations among HL, sociodemographic factors, therapeutic adherence, and the ability to self-manage care. However, such studies predominantly focus on elective or longitudinal follow- up scenarios, with scientific production focused on urgent and emergency services remaining limited.

Within the context of emergency services, the literature includes research conducted with caregivers in pediatric emergency departments^(17)^, comparative analyses with the general population^(18)^, and studies addressing factors related to self- management in hospital emergency settings^(19)^. However, there is a scarcity of evidence directed at intermediate emergency services, such as 24-hour *UPA*, which occupy a strategic position in the Emergency Care Network and are characterized by high demand for care, communicative vulnerability, and the need for rapid decisions. In this scenario, limitations in HL can compromise the understanding of health guidelines, adherence to treatment, and the appropriate use of different points in the healthcare network. Thus, analyzing HL of the users of the 24-hour *UPA* and its relationship with sociodemographic and clinical characteristics contributes to reducing the knowledge gap and improving healthcare practice. This study is based on the hypothesis that 24-hour *UPA’*s users present levels of HL associated with sociodemographic, educational, behavioral, and clinical characteristics, which are linked to understanding of instructions, adherence to treatment, and the pattern of use of health services.

In this context, this study aims to evaluate health literacy and its sociodemographic and clinical associations among users of an emergency care unit.

## METHOD

### Design of Study and Local

Cross-sectional study, according to STROBE guidelines, conducted at a type I 24-hour *UPA* in Rio Grande do Sul, Brazil, which performs an average of 5,651 consultations per month. The *UPA* has facilities for adult and pediatric care, operating 24 hours a day, seven days a week, with a minimum team consisting of doctors, nurses, and nursing technicians, in addition to laboratory and radiological support.

### Sample Definition

The sample included users aged 18 or older who were seen between January and February 2024. Individuals with cognitive impairments identified as disorientation, incoherence, or inability to understand information, as well as those unable to communicate verbally or respond to the instrument, were excluded. Cognitive impairment was assessed by a trained nurse prior to data collection. Patients classified as red risk were excluded because these are cases requiring immediate attention, characterized by severe clinical instability, imminent risk of death, and the need for rapid intervention.

The sample size calculation considered a population of 5,651 users, a margin of error of 4%, and a confidence level of 95%, resulting in 543 participants. The sampling was probabilistic, with 1 out of every 10 eligible users being selected after the initial draw. The following formula was used:


$${\text{n = [N · Z}}^{\text{2}}{\text{· p (1-p)]/[Z}}^{\text{2}}{\text{· p (1-p) + e}}^{\text{2}}\text{(N-1)]}$$

### Data Collection

The participants were invited to the study upon their admission to the *UPA*. The sociodemographic questionnaire included age, sex, marital status, cohabitation, family income, participant*’*s education level, and parents*’* education level (no education/don*’*t know, elementary school, high school, or higher education/postgraduate). Behavioral and health condition variables were also collected: reading habits, assessed by a dichotomous question (“Do you have a reading habit?”), with “yes” indicated when there was routine reading of printed or digital materials; and adherence to treatment, investigated by self-report (“Do you regularly follow the prescribed guidelines and treatments?”), with “yes” indicated when the individual complied with most of the recommendations. The presence and type of health problem, service requests in the last 30 days, and sources of information were also recorded. The collection took place in person via *Google Forms*. HLQ, validated in Brazil^(7–8)^, assessed HL using scales of 1 to 5, according to original recommendations^(8)^. Its use was defined by conducting a multidimensional assessment. Although the HLQ includes nine scales covering individual, interpersonal, and organizational dimensions of HL, this study prioritized scales 1 to 5 because they represent competencies directly mobilized during care in the *UPA*, such as understanding professional guidance, evaluating the information received, and engaging in care.

### Data Analysis and Treatment

Statistical analysis was performed using IBM SPSS 25.0, adopting a significance level of 5%. Descriptive analyses, Kolmogorov–Smirnov normality test, and comparisons between groups using Student*’*s t-test or one-way ANOVA with Bonferroni post-hoc test were performed. Furthermore, the HLQ scales were compared according to education level, parental education level, reading habits, presence and type of health problem, use of health services in the past 30 days, source of information, and adherence to treatment, employing the same tests as applicable. In the comparative analysis, family income was categorized into three ranges: up to R$ 2,900.00; above R$ 2,900.00 up to R$ 4,900.00; and above R$ 4,900.00.

### Ethical Aspects

The use of HLQ was authorized by the copyright holders (Swinburne University) (hlinfo@swin.edu.au) and the project was approved by the Research Ethics Committee (CAAE No. 75589123800005350, Opinion No. 6570946, December 11, 2023). Data collection was carried out with the consent of the participant(s) in the Free Informed Consent Form (FICF).

## RESULTS

A total of 550 users of the 24-hour *UPA* participated, mostly women (54.5%) and adults between 18 and 40 years old (54.5%). The majority of individuals were white (71.6%), had high school education (52.3%), and lived with other people (86.9%). The distribution according to marital status was balanced, while monthly income showed a relatively homogeneous distribution among the three income ranges analyzed (Table 1).

**Table 1:** Sociodemographic and health condition characteristics of users accessing the 24-hour *UPA* – Ijuí, RS, Brazil, 2024.

Variables	Categories	n	%
Sex	Female	300	54.5%
	Male	250	45.5%
Age	18 to 40 years	300	54.5%
	Over 40 years	250	45.5%
Race/color	White	394	71.6%
	Non-white^*^	156	28.4%
Marital status	Lives together	272	49.4%
	Does not live together	278	50.6%
Cohabitation	Lives with someone	478	86.9%
	Lives alone.	72	13.1%
Level of education	Up to high school	469	85.3%
	Undergraduate or graduate degree	81	14.7%
Family income (R$)	Up to 2,900.00	185	33.6%
	Above 2,900.00 up to 4,900.00	180	32.7%
	Above 4,900.00	180	32.7%

Note: *Non-white: those who identify themselves as yellow, brown, and black.

Analysis of patients*’* health conditions indicated that half of the users did not report any chronic disease (303; 55.1%) and the remainder (247; 44.9%) reported that they were affected by various pathologies, such as diabetes mellitus (9; 1.6%), systemic arterial hypertension – SAH (81; 14.7%), concomitant diabetes mellitus and SAH (24; 4.3%), mental disorder (56; 10.1%), among other health conditions. The HL assessment identified “social support for health” and “evaluation of health information” as strengths, and the other scales as weaknesses (Table 2), considering the highest averages observed, even with slight differences between the scales.

**Table 2:** Average health literacy levels of users accessing the 24-hour *UPA* according to scales 1 to 5 of the Health Literacy Questionnaire – Ijuí, RS, Brazil, 2024.

Scales	Mean	Standard deviation
1. Understanding and support from healthcare professionals	2.69	0.58
2. Sufficient information to take care of their health	2.64	0.49
3. Active health care	2.69	0.43
4. Social support for health	2.95	0.60
5. Evaluation of health information	2.71	0.44

HL varied according to sociodemographic characteristics. Significant variation was observed in HL according to sex, age, marital status, cohabitation, and income. Women showed higher averages in the information evaluation domain (p = 0.021). Users over 40 years of age exhibited higher levels of comprehension, active caregiving, and social support (p < 0.05). Living together was associated with higher scores in information and information assessment. Cohabitation influenced social support (p = 0.008). Higher income showed better performance in social support and information assessment (p < 0.01), indicating socioeconomic influence on literacy. (Table 3).

**Table 3:** Average health literacy levels of users accessing the 24-hour UPA, according to sociodemographic characteristics – Ijuí, RS, Brazil, 2024.

Sociodemographic characteristics demográficas	HLQ										
	n	E1		E2		E3		E4		E5	
		Mean	SD	Mean	SD	Mean	SD	Mean	SD	Mean	SD
**Sex**										
Male	250	2.64	0.59	2.60	0.47	2.68	0.44	2.66	0.61	2.67	0.47
Female	300	2.73	0.57	2.66	0.48	2.75	0.42	2.75	0.63	2.75	0.41
p (value)^*^	0.061			0.122		0.837		0.169		0.021	
**Age range**										
18 to 40 years	300	2.57	0.57	2.60	0.48	2.64	0.42	2.91	0.60	2.70	0.41
Over 40 years	250	2.84	0.56	2.67	0.50	2.75	0.44	3.01	0.57	2.71	0.48
p (value)^*^	< 0.001			0.175		0.002		0.044		0.129	
**Marital status**										
Lives together	272	2.72	0.57	2.68	0.48	2.72	0.42	3.00	0.60	2.76	0.44
Does not live together	278	2.66	0.59	2.60	0.49	2.65	0.44	2.91	0.59	2.67	0.43
p (value)^*^	0.186			0.045		0.051		0.082		0.010	
**Cohabitation**										
Lives alone	72	2.75	0.56	2.64	0.53	2.67	0.42	2.78	0.65	2.70	0.45
Lives with someone	478	2.68	0.58	2.64	0.48	2.69	0.43	2.98	0.59	2.72	0.44
p (value)^*^	0.340			0.999		0.719		0.008		0.844	
**Income**										
Up to R$ 2,900.00	185	2.67	0.61	2.60	0.49	2.64	0.43	2.87b	0.66	2.64b	0.45
From R$2,900.00 to R$4,900.00	180	2.68	0.53	2.62	0.47	2.69	0.42	2.93a,b	0.58	2.71a,b	0.39
Above R$ 4,900.00	185	2.72	0.60	2.70	0.50	2.73	0.44	3.06a	0.54	2.79a	0.47
p (value)^*^	0.677			0.114		0.185		0.006		0.007	

Note: HLQ – Health Literacy Questionnaire; E1. Understanding and support from healthcare professionals; E2. Sufficient information to take care of their health; E3 Active health care; E4. Social support for health; E5. Evaluation of health information; SD – Sample standard deviation. *Student’s t-test for independent groups; **Analysis of Variance (One-way).

Table 4 shows that HL levels were significantly higher in users with higher education or postgraduate degrees, especially in understanding and support from professionals, access to sufficient information, active care, and evaluation of information (p < 0.05). The mother*’*s and father*’*s education level was also positively correlated with HL literacy, highlighting the influence of parents on health information understanding and assessment. No significant differences in social support were observed according to education level.

**Table 4:** Average health literacy levels of users accessing the 24-hour UPA, according to their level of education and that of their parents – Ijuí, RS, Brazil, 2024.

Levels of education	HLQ										
	n	E1		E2		E3		E4		E5	
		Mean	SD	Mean	SD	Mean	SD	Mean	SD	Mean	SD
**Level of education**										
Elementary education	181	2.73^a,b^	0.55	2.56^b^	0.45	2.66^b^	0.42	2.59	0.44	2.63^b^	0.44
High school	288	2.62^b^	0.60	2.62^b^	0.51	2.67^b^	0.43	2.69	0.44	2.73^b^	0.44
Undergraduate or graduate degree	81	2.83^a^	0.56	2.87^a^	0.43	2.80^a^	0.47	2.84	0.41	2.84^a^	0.41
p(value)^*^	0.010			< 0.001		0.042		0.126		0.001	
**Mother’s education level**										
No instruction or does not know	156	2.79^a^	0.55	2.64	0.49	2.71	0.47	3.02	0.54	2.60c	0.46
Elementary education	281	2.62^b^	0.61	2.63	0.50	2.69	0.43	2.95	0.64	2.74^b^	0.46
High school	79	2.68^b^	0.51	2.64	0.45	2.63	0.37	2.91	0.49	2.76^b^	0.33
Undergraduate or graduate degree	34	2.77^a^	0.62	2.76	0.48	2.72	0.42	2.79	0.65	2.89^a^	0.25
p(value)^*^	0.023			0.541		0.603		0.198		0.001	
**Father’s education level**										
No instruction or does not know	191	2.77^a^	0.60	2.61	0.51	2.66	0.46	3.01	0.55	2.62^b^	0.48
Elementary education	265	2.62^b^	0.58	2.64	0.48	2.70	0.42	2.88	0.67	2.74^a,b^	0.43
High school	67	2.71^a,b^	0.51	2.68	0.50	2.70	0.38	3.06	0.41	2.81ª	0.38
Undergraduate or graduate degree	27	2.79^a^	0.56	2.76	0.38	2.73	0.47	3.01	0.47	2.82ª	0.27
p(value)^*^	0.038			0.395		0.837		0.060		0.003	

Note: HLQ – Health Literacy Questionnaire; E1. Understanding and support from healthcare professionals; E2. Sufficient information to take care of their health; E3 Active health care; E4. Social support for health; E5. Health information assessment; SD – Sample standard deviation; *Analysis of Variance (One way); a and/or b Post Hoc Bonferroni: compared the means of the studied variable among the groups; if the means were marked with the same superscript letters, they do not show a statistically significant difference, but they show significance if they were marked with different superscript letters.

Table 5 shows that users with a reading habit had better scores in all dimensions of the HLQ (p < 0.005), indicating a higher HL. Health problems were associated with higher scores in E1 and E2 (p < 0.05), and those with multiple conditions showed greater social support (E4) (p < 0.001). Adherence to treatment was linked to better outcomes in almost all dimensions (p < 0.05). Users who sought care in the past 30 days had higher scores in E1 and E2 (p < 0.05), highlighting the “other” group in E1 and E3. Seeking information from professionals increased scores in E1 (p = 0.001). These findings reinforce the importance of reading habits and adherence to treatment for HL in the 24-hour *UPA*.

**Table 5:** Average health literacy levels of users accessing the 24-hour UPA, according to their habits and health conditions – Ijuí, RS, Brazil, 2024.

Health conditions	HLQ										
	n	E1		E2		E3		E4		E5	
		Mean	SD	Mean	SD	Mean	SD	Mean	SD	Mean	SD
**Reading habit**										
Yes	253	2.82	0.55	2.72	0.46	2.75	0,41	3.03	0.56	2.80	0.42
No	297	2.58	0.58	2.57	0.50	2.63	0.45	2.89	0,62	2.64	0.45
p (value)^*^		< 0.001		< 0.001		0.001		0.005		< 0.001	
**Health Problem**										
Yes	247	2.82	0.56	2.69	0.50	2.72	0.44	2,99	0,65	2.74	0.44
No	303	2.58	0.58	2.60	0.48	2.66	0.42	2,93	0,55	2.69	0.44
p (value)^*^		< 0.001		0.023		0.130		0.239		0.139	
**Which health problems**										
DM	9	2.78	0.36	2.50^c^	0.35	2.73	0,32	3,13^b^	0,85	2.51	0.40
SAH	81	2.79	0.43	2.62^bc^	0.42	2.71	0.27	2.93^bb^	0.58	2.74	0.36
DM + SAH	24	2.92	0.25	2.68^b^	0.34	2.79	0.24	2.98^a,b^	0.47	2.56	0.33
SM	56	2.70	0.61	2.62^b^	0.48	2.63	0.42	2.73^c^	0.74	2.80	0.34
Others^‡^	77	2.92	0.69	2.85^a^	0.61	2.76	0.62	3.22^a^	0.62	2.80	0.59
p (value)^*^		0.186		0.018		0.491		< 0.001		0.066	
**Adherence to Health Treatment**										
Yes	215	2.85	0.57	2.72	0.49	2.75	0.45	3.02	0.63	2.98	0.77
No, or sometimes	335	2.63	0.43	2.54	0.53	2.55	0.36	2.77	0.76	2.85	0.62
p (value)^*^		0.023		0.046		0.009		0.027		0.106	
**Sought any healthcare services in the past 30 days?**										
Yes	258	2.74	0.57	2.70	0.49	2.69	0.44	2.96	0.64	2.73	0.45
No	292	2.64	0.59	2.59	0.49	2.68	0.43	2.95	0.56	2.70	0.43
p (value)^*^		0.037		0.01		0.863		0.868		0.309	
**Which service sought**										
*UPA*	96	2.64^b^	0.48	2.68	0.43	2.59^b^	0,38	2.89	0,64	2.95	0.64
PHC	138	2.77^a,b^	0.61	2.68	0.52	2.71^a,b^	0,42	2.96	0,65	2.73	0.35
Others^†^	19	3.29^a^	0.43	2.74	0.66	3.06^a^	0,61	3.19	0,74	2.99	0.76
p (value)^**^	0.003			0.137		0.014		0.524		0.055	
**Information search**										
With healthcare professionals										
No	248	2.59	0.59	2.62	0.51	2.65	0.44	2.96	0.57	2.71	0.45
Yes	302	2.77	0.56	2.66	0.47	2.72	0.42	2.95	0.62	2.71	0.43
p (value)^*^		0.001		0.331		0.079		0.891		0.954	

Note: HLQ – Health Literacy Questionnaire; E1. Understanding and support from healthcare professionals; E2. Sufficient information to take care of their health; E3 Active health care; E4. Social support for health; E5. Assessment of health information; DM – Diabetes Mellitus; SAH – Systemic Arterial Hypertension; MH – mental health problems such as depression and anxiety; *UPA* – 24-hour Emergency Care Unit; PHC – Primary Health Care; SD – Sample standard deviation; *Student’s t-test for two independent groups;

**Analysis of Variance (One way); a and/or b and/or c Post Hoc Bonferroni, compared the means of the studied variable between the groups; if the means were marked with the same superscript letters, they do not show a statistically significant difference, but they show significance if they were marked with different superscript letters;

‡Others – Chronic Obstructive Pulmonary Disease, asthma, renal failure, heart disease, stroke, neoplasm, Parkinson’s, Alzheimer’s, rheumatological diseases, obesity;

†Others – Private practice, Psychosocial Care Centers (CAPS). One user reported having used all the services.

## DISCUSSION

This pioneering study in Brazil evaluated the HL of users of a 24-hour *UPA* and identified a profile marked by sociodemographic and clinical inequalities, with direct implications for communication, decision-making, and continuity of care in emergency settings. The greatest strengths lay in social support for health and in the evaluation of information, while the main weaknesses were observed in the understanding and support of professionals, access to sufficient information, and active health care. This pattern is consistent with international findings indicating lower HL in emergency services compared to general populations, as observed in France and Germany^(17,19)^.

The social support dimension showed the highest averages, aligning with evidence that support networks positively influence well-being, therapeutic adherence, and the ability to cope with acute situations^(20)^. Similarly, the positive evaluation of information suggests that users are able to discriminate between reliable content, a finding also reported among Japanese adults, who demonstrated informational judgment skills relevant to self-care^(21)^.

HL varied significantly according to sociodemographic characteristics, reinforcing its association with social determinants of health. Women performed better in assessing the information, a result supported by another study that also suggests greater female initiative in seeking and interpreting health data^(22)^. However, the literature highlights the need to strengthen women*’*s HL, given their central role in managing family care^(23)^.

People over 40 years old had higher scores in comprehension, professional support, active caregiving, and social support. International studies suggest that this advantage may stem from greater familiarity with healthcare services and prior experience in managing chronic conditions^(24)^.

Cohabitation showed a positive association with social support and information gathering and evaluation skills, reinforcing the contribution of affective and social networks, as pointed out in studies that highlight the role of emotional and instrumental support in HL^(18,25)^. Family income primarily influenced social support and information assessment, a finding consistent with research identifying lower HL among low-income individuals due to inequalities in access to educational resources, technologies, and services^(26-27)^.

The individual*’*s own education level and that of their parents emerged as central determinants of HL, influencing domains related to comprehension, sufficient information, and information evaluation. Evidence shows that low levels of education limit the understanding of guidelines and forms, hindering decision-making and navigation within the healthcare system^(28)^. The strong relationship between parental education and HL reinforces the intergenerational transmission of educational capital and cognitive skills relevant to caregiving.

The reading habit was one of the behavioral factors most strongly associated with HL, corroborating studies that show that regular reading favors the development of cognitive and interpretive skills used in understanding instructions and managing health^(29)^. This finding highlights opportunities for educational interventions aimed at promoting reading practices as a strategy for strengthening HL.

Users with health problems showed higher levels of limited HL compared to those without illnesses, contrasting with French data that showed higher limited HL among patients with chronic conditions^(19)^. This difference can be explained by these users*’* greater contact with the healthcare system, although it is recognized that diseases such as hypertension and diabetes can impair cognition and negatively influence HL^(30)^.

Adherence to treatment was associated with better scores across several dimensions of HL, consistent with evidence linking adequate HL to understanding prescriptions, effective self-care, and safe decision-making^(31)^. Users who sought healthcare services in the last 30 days performed better in obtaining information, suggesting that recent contact with professionals may reinforce guidance and promote greater clarity in procedures^(32)^.

Seeking information from professionals was also associated with greater understanding and perceived support, underscoring the fundamental role of professional-user communication. Strategies such as Teach-back, which are widely recommended, help verify comprehension and reduce communication failures^(33)^. Considering that HL is conceived as a lightweight technology anchored in the connection, trust, and adaptation of language to the user*’*s context^(3)^, the nursing professional plays a strategic role in reducing informational inequalities.

Finally, it is observed that the frequent use of the 24-hour *UPA* may reflect limitations in HL, expressed by difficulties in adherence to treatment, in navigating the different points of the care network, and in the proper interpretation of health guidelines, which results in an overload of services and increased healthcare costs^(34)^. In this regard, strengthening HL is a fundamental strategy for reducing unnecessary demands, preventing clinical decompensation, supporting informed decisions, and promoting greater equity in access to care^(19)^. Moreover, it is essential that healthcare professionals recognize the uniqueness of each user and incorporate HL strategies into their care practice, promoting the empowerment of individuals and expanding their capacity to actively participate in decisions related to their own health and well-being^(35)^.

These findings reinforce the HL potential as a strategic tool to improve the organization of care in the *UPA*, contributing to safer decisions, effective communication, and integration with the Emergency Care Network.

Among the study limitations, the exclusion of users classified as red risk and those with cognitive impairment stands out. For ethical and clinical reasons, they could not be included, which may restrict the understanding of HL in more vulnerable groups. Furthermore, the cross-sectional design and the fact that the research was conducted in a single type I *UPA* limit the generalizability of the results. It is recommended that future investigations incorporate strategies for including more complex clinical profiles, broaden the representativeness of participants, and consider conducting multivariate analyses to deepen the understanding of factors associated with HL in this context.

## CONCLUSION

This study demonstrated that the HL of users of a 24-hour *UPA* varies significantly according to sociodemographic and clinical factors, particularly sex, age, marital status, income, education level, reading habits, and adherence to treatment. The greatest strengths identified were social support and information assessment, while weaknesses related to communication with professionals, access to sufficient information, and proactive health management remained. The findings highlight the usefulness of HLQ in identifying patterns of HL in this healthcare context, contributing to an understanding of the informational vulnerabilities present in emergency services.

## Data Availability

The entire dataset supporting the results of this study is available upon request to the corresponding author.
