# Supplementary material for: Health literacy and its sociodemographic and health determinants
Source: Rev Esc Enferm USP. 2026 Jun 12;60:e20250441. doi: 10.1590/1980-220X-REEUSP-2025-0441en (PMC13262926; doi:10.1590/1980-220X-REEUSP-2025-0441en)
Supplement: Translation

# Translation: Letramento em saúde e seus determinantes sociodemográficos e de saúde

**DOI:** 10.1590/1980-220X-REEUSP-2025-0441pt

**Authors:** Cristiane Ribas, Christiane de Fátima Colet, Edgar Noé Morelos-Garcia, Olga Maria Martins de Sousa Valentim, Márcio Strassburger, Fernanda Dal Maso Camera, Carolina Rodrigues Rusch, Adriane Cristina Bernat Kolankiewicz

**Affiliations:** 1 Universidade Regional do Noroeste do Estado do Rio Grande do Sul, Programa de Pós-Graduação Strictu Sensu em Atenção Integral à Saúde, Ijuí, RS, Brasil.; 2 Universidade Autônoma de Tamaulipas, Tamaulipas, Mexico.; 3 Universidade de Lisboa, Escola Superior de Enfermagem de Lisboa, Centro de Investigação, Inovação e Desenvolvimento em Enfermagem de Lisboa, Lisboa, Portugal.; 4 Universidade do Porto, Escola Superior de Enfermagem, RISE-Health, Porto, Portugal.; 5 Universidade Regional do Noroeste do Estado do Rio Grande do Sul, Curso de Fisioterapia, Ijuí, RS, Brasil.; 6 Universidade Regional Integrada do Alto Uruguai e das Missões, Programa de Pós-Graduação Strictu Sensu em Atenção Integral à Saúde, Erechim, RS, Brasil.; 7 Universidade Regional do Noroeste do Estado do Rio Grande do Sul, Curso de Enfermagem, Ijuí, RS, Brasil.

**Keywords:** Serviços Médicos de Emergência, Literacia para a Saúde, Comportamentos Relacionados com a Saúde, Características da População.

## Abstract

**Objetivo:**

Avaliar o letramento em saúde e suas associações sociodemográficas e clínicas entre usuários de unidade de pronto atendimento.

**Método:**

Estudo transversal realizado em unidade de pronto atendimento 24h no sul do Brasil, com amostra de 550 usuários (≥18 anos). Aplicaram-se questionário sociodemográfico e clínico e as escalas 1 a 5 do Health Literacy Questionnaire. Realizaram-se análises descritivas e inferenciais (teste t de Student e ANOVA; p ≤ 0,05).

**Resultados:**

As maiores médias ocorreram em “suporte social” (2,95 ± 0,60) e “avaliação das informações” (2,71 ± 0,44), e as menores em “informações suficientes” (2,64 ± 0,49) e “compreensão profissional” (2,69 ± 0,58). Escores mais elevados associaram-se ao sexo feminino (p = 0,021), idade >40 anos (p ≤ 0,044), viver em união (p = 0,045; p = 0,010), coabitação (p = 0,008), maior renda (p = 0,006; p = 0,007) e maior escolaridade própria e parental (p ≤ 0,038). Hábito de leitura associou-se a todas as dimensões (p ≤ 0,005). Problema de saúde (p ≤ 0,023), adesão ao tratamento (p < 0,05), procura recente de serviço (p ≤ 0,037) e busca de informações com profissionais (p = 0,001) também influenciaram os escores.

**Conclusão:**

O letramento em saúde variou significativamente segundo características sociodemográficas e clínicas entre usuários.

## INTRODUÇÃO

O Sistema Único de Saúde (SUS) estrutura-se em três bases hierárquicas, considerando os níveis de atenção à saúde: Atenção Básica, média complexidade e alta complexidade. A articulação desses níveis é essencial para um atendimento adequado e eficiente, visando o cuidado integral do paciente^(1)^. Como integrante dos serviços de saúde do SUS, a Unidade de Pronto Atendimento 24h (UPA 24h) é um serviço com complexidade intermediária entre a Atenção Primária à Saúde (APS) e a Rede Hospitalar. O serviço visa garantir atendimento resolutivo aos usuários acometidos por quadros agudos de natureza clínica e em situações cirúrgicas e trauma^(2)^.

Neste contexto, o Letramento em Saúde (LS) é essencial para que a população possa cuidar de sua saúde, fazer o acompanhamento, aderir ao tratamento e tomar decisões em prol de sua saúde, o que pode impactar diretamente na qualidade de saúde dos indivíduos e da comunidade^(3)^. Em 2020, o *U.S. Department of Health and Human Services* (Departamento de Saúde e Serviços Humanos do Estados Unidos, em português) definiu LS – *Health Literacy* como “o grau de habilidade que cada indivíduo tem para encontrar, compreender e utilizar informações e serviços para tomar decisões e ações para a própria saúde e de outros”^(4)^.

De forma convergente, a Organização Mundial de Saúde ampliou a definição ao afirmar que o LS não é apenas um atributo individual, mas também um produto das condições sociais, econômicas e institucionais. Assim, cabe aos serviços de saúde criar ambientes, materiais e práticas comunicacionais que apoiem a compreensão, a tomada de decisão e o exercício da autonomia, destacando o papel das organizações de saúde no desenvolvimento das habilidades de LS da população^(5)^.

O LS tem múltiplos conceitos e está relacionado e interposto entre os campos da saúde e da educação, visando melhoria da qualidade de vida e autonomia do indivíduo. Para avaliar as potencialidades e fragilidades do LS dos indivíduos^(6)^, uma das ferramentas utilizadas é o *Health Literacy questionnaire* (HLQ)^(7)^. Trata-se de um instrumento multidimensional, aplicável em diferentes culturas e países com intuito de avaliar o LS, para além das habilidades cognitivas de leitura e numeramento^(7–8)^.

Estudos sobre LS têm sido conduzidos em diferentes contextos assistenciais e populacionais, incluindo usuários da APS com condições crônicas, como hipertensão arterial e diabetes mellitus tipo II^(9)^, bem como indivíduos submetidos à terapia dialítica^(10)^, pessoas na emergência^(11)^, com doença arterial coronariana^(12)^, pacientes com câncer hematológico^(13)^, profissionais de saúde^(14)^, cuidadores domiciliares^(15)^ e pessoas que vivem com transtornos mentais graves e persistentes^(16)^. De modo geral, essas investigações evidenciam associações consistentes entre o LS, fatores sociodemográficos, adesão terapêutica e capacidade de autogestão do cuidado. Entretanto, tais estudos concentram-se predominantemente em cenários eletivos ou de acompanhamento longitudinal, permanecendo limitada a produção científica voltada aos serviços de urgência e emergência.

No âmbito dos serviços de emergência, a literatura contempla investigações realizadas com cuidadores em pronto-socorro pediátrico^(17)^, análises comparativas com a população geral^(18)^ e estudos que abordam fatores relacionados à autogestão em contextos hospitalares de emergência^(19)^. Entretanto, observa- se escassez de evidências direcionadas aos serviços de urgência intermediários, como as UPA 24h, que ocupam posição estratégica na Rede de Atenção às Urgências e caracterizam-se por elevada demanda assistencial, vulnerabilidade comunicacional e necessidade de decisões rápidas. Nesse cenário, limitações no LS podem comprometer a compreensão das orientações em saúde, a adesão ao tratamento e o uso adequado dos diferentes pontos da rede assistencial. Assim, analisar o LS de usuários da UPA 24h e sua relação com características sociodemográficas e clínicas contribui para reduzir a lacuna do conhecimento e qualificar a prática assistencial. Parte-se, neste estudo, da hipótese de que usuários da UPA 24h apresentam níveis de LS associados a características sociodemográficas, educacionais, comportamentais e clínicas, os quais se associam à compreensão das orientações, a adesão ao tratamento e o padrão de utilização dos serviços de saúde.

Nesse contexto, este estudo visa avaliar o letramento em saúde e suas associações sociodemográficas e clínicas entre usuários de unidade de pronto atendimento.

## MÉTODO

### Tipo e Local do Estudo

Estudo transversal, conforme *guidelines* STROBE, realizado em uma Unidade de Pronto Atendimento (UPA) 24h de porte I no Rio Grande do Sul, Brasil, que realiza em média 5.651 atendimentos mensais. A UPA dispõe de estrutura para atendimento adulto e pediátrico, funcionando 24 horas, sete dias por semana, com equipe mínima composta por médicos, enfermeiros e técnicos de enfermagem, além de suporte laboratorial e radiológico.

### Definição da Amostra

A amostra incluiu usuários ≥18 anos atendidos entre janeiro e fevereiro de 2024. Foram excluídos indivíduos com déficit cognitivo identificado por desorientação, incoerência ou incapacidade de compreender informações, bem como aqueles impossibilitados de se comunicar verbalmente ou responder ao instrumento. O déficit cognitivo foi avaliado por um enfermeiro treinado, previamente à coleta de dados. Foram excluídos os pacientes classificados como risco vermelho, por se tratarem de casos que exigem atendimento imediato, caracterizados por instabilidade clínica grave, risco iminente de morte e necessidade de intervenção rápida.

O cálculo amostral considerou população de 5.651 usuários, erro de 4% e confiança de 95%, resultando em 543 participantes. A amostragem foi probabilística, selecionando-se 1 a cada 10 usuários elegíveis, após sorteio do ponto inicial. Utilizou-se a fórmula:


$${\text{n = [N · Z}}^{\text{2}}{\text{· p (1-p)]/[Z}}^{\text{2}}{\text{· p (1-p) + e}}^{\text{2}}\text{(N-1)]}$$

### Coleta de Dados

Os participantes foram convidados a participar do estudo a partir do seu ingresso na UPA. O questionário sociodemográfico incluiu idade, sexo, estado civil, coabitação, renda familiar, escolaridade do participante e escolaridade dos pais (nenhuma instrução/não sabe, ensino fundamental, ensino médio ou ensino superior/pós-graduação). Também foram coletadas variáveis comportamentais e de condições de saúde: hábito de leitura, avaliado por pergunta dicotômica (“Você possui hábito de leitura?”), sendo sim quando havia leitura rotineira de materiais impressos ou digitais; e adesão ao tratamento, investigada por autorrelato (“Você segue regularmente as orientações e tratamentos prescritos?”), considerando-se sim quando o indivíduo cumpria a maior parte das recomendações. Registraram-se ainda presença e tipo de problema de saúde, procura de serviços nos últimos 30 dias e fonte de busca de informações. A coleta ocorreu presencialmente via *Google Forms*. O HLQ, validado no Brasil ^(7–8)^, avaliou o LS pelas escalas 1 a 5, conforme recomendações originais^(8)^. Seu uso foi definido por realização de uma avaliação multidimensional. Embora o HLQ contemple nove escalas que abrangem dimensões individuais, interpessoais e organizacionais do LS, este estudo priorizou as escalas 1 a 5 por representarem competências diretamente mobilizadas no momento do atendimento na UPA, como a compreensão das orientações profissionais, a avaliação das informações recebidas e o engajamento no cuidado.

### Análise e Tratamento dos Dados

A análise estatística foi realizada no IBM SPSS 25.0, adotando significância de 5%. Realizaram-se análises descritivas, teste de normalidade de Kolmogorov–Smirnov e comparações entre grupos por t de Student ou ANOVA One-Way com post-hoc Bonferroni. Além disso, compararam-se as escalas do HLQ segundo escolaridade, escolaridade dos pais, hábito de leitura, presença e tipo de problema de saúde, procura de serviços de saúde nos últimos 30 dias, fonte de busca de informações e adesão ao tratamento, empregando os mesmos testes conforme aplicável. Na análise comparativa, a renda familiar foi categorizada em três faixas: até R$ 2.900,00; acima de R$ 2.900,00 até R$ 4.900,00; e acima de R$ 4.900,00.

### Aspectos Éticos

O uso do HLQ foi autorizado pelos detentores dos direitos autorais (Swinburne University) (hlinfo@swin.edu.au) e projeto aprovado pelo Comitê de Ética em Pesquisa (CAAE nº 75589123800005350, Parecer nº 6570946, 11 de dezembro de 2023). A coleta dos dados ocorreu mediante anuência no Termo de Consentimento Livre Esclarecido (TCLE), pela(o)s participante(s).

## RESULTADOS

Participaram 550 usuários da UPA 24h, majoritariamente mulheres (54,5%) e adultos entre 18 e 40 anos (54,5%). Predominaram indivíduos brancos (71,6%), com ensino médio (52,3%) e coabitando com outras pessoas (86,9%). A distribuição segundo estado civil mostrou-se equilibrada, enquanto a renda mensal apresentou distribuição relativamente homogênea entre as três faixas de renda analisadas (Tabela 1).

**Tabela 1:** Caracterização sociodemográfica e de condições de saúde dos usuários que acessam a UPA 24h- Ijuí, RS, Brasil, 2024.

Variáveis	Categorias	n	%
Sexo	Feminino	300	54,5%
	Masculino	250	45,5%
Idade	18 a 40 anos	300	54,5%
	Acima de 40 anos	250	45,5%
Raça/cor	Branca	394	71,6%
	Não branca^*^	156	28,4%
Estado civil	Vivem em união	272	49,4%
	Não vivem em união	278	50,6%
Coabitação	Vive com alguém	478	86,9%
	Vive sozinho	72	13,1%
Escolaridade	Até ensino médio	469	85,3%
	Superior/pós-graduação	81	14,7%
Renda familiar (R$)	Até 2.900,00	185	33,6%
	Acima de 2.900,00 até 4.900,00	180	32,7%
	Acima de 4.900,00	180	32,7%

Nota: *Não branca: aqueles que se identificam como amarelos, pardos e pretos.

A análise das condições de saúde dos pacientes indicou que metade dos usuários não relatou nenhuma doença crônica (303; 55,1%) e os demais (247; 44,9%) informaram que eram acometidos por diversas patologias, como diabetes mellitus (9; 1,6%), hipertensão arterial sistêmica – HAS (81; 14,7%), diabetes mellitus e HAS concomitantes (24; 4,3%), transtorno mental (56; 10,1%), entre outras condições de saúde. A avaliação do LS identificou como pontos fortes o “suporte social para a saúde” e a “avaliação das informações em saúde” e como fragilidades as demais escalas (Tabela 2), considerando as maiores médias observadas, ainda que com diferenças discretas entre as escalas.

**Tabela 2:** Médias de letramento em saúde dos usuários que acessam a UPA 24h referente às escalas 01 a 05 do Health Literacy Questionnaire- Ijuí, RS, Brasil, 2024.

Escalas	Média	Desvio Padrão
1. Compreensão e apoio dos profissionais de saúde	2,69	0,58
2. Informações suficientes para cuidar da saúde	2,64	0,49
3. Cuidado ativo da saúde	2,69	0,43
4. Suporte social para saúde	2,95	0,60
5. Avaliação das informações em saúde	2,71	0,44

O LS variou de acordo com as características sociodemográficas. Observou-se variação significativa no LS segundo sexo, idade, estado civil, coabitação e renda. Mulheres apresentaram maiores médias no domínio de avaliação da informação (p = 0,021). Usuários acima de 40 anos exibiram níveis superiores em compreensão, cuidado ativo e suporte social (p < 0,05). Viver em união associou-se a maiores escores em informações e avaliação das informações. A coabitação influenciou o suporte social (p = 0,008). Renda mais alta mostrou melhor desempenho em suporte social e avaliação das informações (p < 0,01), indicando influência socioeconômica no letramento. (Tabela 3).

**Tabela 3:** Médias de letramento em saúde dos usuários que acessam a UPA 24h, segundo as características sociodemográficas – Ijuí, RS, Brasil, 2024.

Características socio demográficas	HLQ										
	n	E1		E2		E3		E4		E5	
		Média	DP	Média	DP	Média	DP	Média	DP	Média	DP
**Sexo**										
Masculino	250	2,64	0,59	2,60	0,47	2,68	0,44	2,66	0,61	2,67	0,47
Feminino	300	2,73	0,57	2,66	0,48	2,75	0,42	2,75	0,63	2,75	0,41
p (value)^*^	0,061			0,122		0,837		0,169		0,021	
**Faixa etária**										
18 a 40 anos	300	2,57	0,57	2,60	0,48	2,64	0,42	2,91	0,60	2,70	0,41
Acima de 40 anos	250	2,84	0,56	2,67	0,50	2,75	0,44	3,01	0,57	2,71	0,48
p (value)^*^	< 0,001			0,175		0,002		0,044		0,129	
**Estado civil**										
Vive em união	272	2,72	0,57	2,68	0,48	2,72	0,42	3,00	0,60	2,76	0,44
Não vive em união	278	2,66	0,59	2,60	0,49	2,65	0,44	2,91	0,59	2,67	0,43
p (value)^*^	0,186			0,045		0,051		0,082		0,010	
**Coabitação**										
Vive sozinho	72	2,75	0,56	2,64	0,53	2,67	0,42	2,78	0,65	2,70	0,45
Vive com alguém	478	2,68	0,58	2,64	0,48	2,69	0,43	2,98	0,59	2,72	0,44
p (value)^*^	0,340			0,999		0,719		0,008		0,844	
**Renda**										
Até R$ 2.900,00	185	2,67	0,61	2,60	0,49	2,64	0,43	2,87b	0,66	2,64b	0,45
Acima de R$ 2.900,00 até R$ 4.900,00	180	2,68	0,53	2,62	0,47	2,69	0,42	2,93a,b	0,58	2,71a,b	0,39
Acima de R$ 4.900,00	185	2,72	0,60	2,70	0,50	2,73	0,44	3,06a	0,54	2,79a	0,47
p (value)^*^	0,677			0,114		0,185		0,006		0,007	

Nota: HLQ – Health Literacy Questionnaire; E1. Compreensão e apoio dos profissionais de saúde; E2. Informações suficientes para cuidar da saúde; E3. Cuidado ativo da saúde; E4. Suporte social para saúde; E5. Avaliação das informações em saúde; DP – Desvio padrão amostral. *Teste t-Student para grupos independentes;

**Análise de Variância (One way).

A Tabela 4 mostra que os níveis de LS foram significativamente maiores em usuários com ensino superior ou pós-graduação, especialmente na compreensão e apoio dos profissionais, acesso a informações suficientes, cuidado ativo e avaliação das informações (p < 0,05). A escolaridade da mãe e do pai também se relacionou positivamente com o LS, destacando-se a influência dos pais na compreensão e avaliação das informações de saúde. Não foram observadas diferenças significativas no apoio social de acordo com a escolaridade.

**Tabela 4:** Médias de letramento em saúde dos usuários que acessam a UPA 24h, segundo o seu nível de instrução e o de seus pais – Ijuí, RS, Brasil, 2024.

Níveis de instrução	HLQ										
	n	E1		E2		E3		E4		E5	
		Média	DP	Média	DP	Média	DP	Média	DP	Média	DP
**Escolaridade**										
Ensino fundamental	181	2,73^a,b^	0,55	2,56^b^	0,45	2,66^b^	0,42	2,59	0,44	2,63^b^	0,44
Ensino médio	288	2,62^b^	0,60	2,62^b^	0,51	2,67^b^	0,43	2,69	0,44	2,73^b^	0,44
Ensino superior ou Pós-graduação	81	2,83^a^	0,56	2,87^a^	0,43	2,80^a^	0,47	2,84	0,41	2,84^a^	0,41
p(value)^*^	0,010			< 0,001		0,042		0,126		0,001	
**Escolaridade da mãe**										
Nenhuma instrução ou não sabe	156	2,79^a^	0,55	2,64	0,49	2,71	0,47	3,02	0,54	2,60^c^	0,46
Ensino fundamental	281	2,62^b^	0,61	2,63	0,50	2,69	0,43	2,95	0,64	2,74^b^	0,46
Ensino médio	79	2,68^b^	0,51	2,64	0,45	2,63	0,37	2,91	0,49	2,76^b^	0,33
Ensino superior ou Pós-graduação	34	2,77^a^	0,62	2,76	0,48	2,72	0,42	2,79	0,65	2,89^a^	0,25
p(value)^*^	0,023			0,541		0,603		0,198		0,001	
**Escolaridade do pai**										
Nenhuma instrução ou não sabe	191	2,77^a^	0,60	2,61	0,51	2,66	0,46	3,01	0,55	2,62^b^	0,48
Ensino fundamental	265	2,62^b^	0,58	2,64	0,48	2,70	0,42	2,88	0,67	2,74^a,b^	0,43
Ensino médio	67	2,71^a,b^	0,51	2,68	0,50	2,70	0,38	3,06	0,41	2,81ª	0,38
Ensino superior ou Pós-graduação	27	2,79^a^	0,56	2,76	0,38	2,73	0,47	3,01	0,47	2,82ª	0,27
p(value)^*^	0,038			0,395		0,837		0,060		0,003	

Nota: HLQ – Health Literacy Questionnaire; E1. Compreensão e apoio dos profissionais de saúde; E2. Informações suficientes para cuidar da saúde; E3. Cuidado ativo da saúde; E4. Suporte social para saúde; E5. Avaliação das informações em saúde; DP – Desvio padrão amostral; *Análise de Variância (One way); a e/ou b Post Hoc Bonferroni: comparou as médias da variável estudada entre os grupos, se a médias foram marcadas com a letras sobrescritas iguais, elas não apresentam diferença estatisticamente significativa, mas apresentam significância se foram marcadas com letras sobrescritas diferentes.

A Tabela 5 mostra que usuários com hábito de leitura apresentaram melhores escores em todas as dimensões do HLQ (p < 0,005), indicando maior LS. Problemas de saúde associaram-se a maiores escores em E1 e E2 (p < 0,05), e aqueles com múltiplas condições mostraram maior suporte social (E4) (p < 0,001). A adesão ao tratamento esteve ligada a melhores resultados em quase todas as dimensões (p < 0,05). Usuários que buscaram atendimento nos últimos 30 dias tiveram escores mais altos em E1 e E2 (p < 0,05), com destaque para o grupo “outros” em E1 e E3. A busca de informações com profissionais elevou os escores em E1 (p = 0,001). Esses achados reforçam a importância do hábito de leitura e da adesão ao tratamento para o LS na UPA 24h.

**Tabela 5:** Médias de letramento em saúde dos usuários que acessam a UPA 24h, segundo seus hábitos e a suas condições de saúde- Ijuí, RS, Brasil, 2024.

Condições de saúde	HLQ										
	n	E1		E2		E3		E4		E5	
		Média	DP	Média	DP	Média	DP	Média	DP	Média	DP
**Hábito de leitura**										
Sim	253	2,82	0,55	2,72	0,46	2,75	0,41	3,03	0,56	2,80	0,42
Não	297	2,58	0,58	2,57	0,50	2,63	0,45	2,89	0,62	2,64	0,45
p (value)^*^	< 0,001			< 0,001		0,001		0,005		< 0,001	
**Problema Saúde**										
Sim	247	2,82	0,56	2,69	0,50	2,72	0,44	2,99	0,65	2,74	0,44
Não	303	2,58	0,58	2,60	0,48	2,66	0,42	2,93	0,55	2,69	0,44
p (value)^*^	< 0,001			0,023		0,130		0,239		0,139	
**Quais problemas de saúde**										
DM	9	2,78	0,36	2,50^c^	0,35	2,73	0,32	3,13^b^	0,85	2,51	0,40
HAS	81	2,79	0,43	2,62^bc^	0,42	2,71	0,27	2,93^bb^	0,58	2,74	0,36
DM + HAS	24	2,92	0,25	2,68^b^	0,34	2,79	0,24	2,98^a,b^	0,47	2,56	0,33
SM	56	2,70	0,61	2,62^b^	0,48	2,63	0,42	2,73^c^	0,74	2,80	0,34
Outros^‡^	77	2,92	0,69	2,85^a^	0,61	2,76	0,62	3,22^a^	0,62	2,80	0,59
p (value)^**^	0,186			0,018		0,491		< 0,001		0,066	
**Adesão ao tratamento de Saúde**										
Sim	215	2,85	0,57	2,72	0,49	2,75	0,45	3,02	0,63	2,98	0,77
Não ou às vezes	335	2,63	0,43	2,54	0,53	2,55	0,36	2,77	0,76	2,85	0,62
p (value)^*^	0,023			0,046		0,009		0,027		0,106	
**Procurou algum Serviço de Saúde nos últimos 30 dias**										
Sim	258	2,74	0,57	2,70	0,49	2,69	0,44	2,96	0,64	2,73	0,45
Não	292	2,64	0,59	2,59	0,49	2,68	0,43	2,95	0,56	2,70	0,43
p (value)^*^	0,037			0,01		0,863		0,868		0,309	
**Qual serviço procurou**										
*UPA*	96	2,64^b^	0,48	2,68	0,43	2,59^b^	0,38	2,89	0,64	2,95	0,64
APS	138	2,77^a,b^	0,61	2,68	0,52	2,71^a,b^	0,42	2,96	0,65	2,73	0,35
Outros^†^	19	3,29^a^	0,43	2,74	0,66	3,06^a^	0,61	3,19	0,74	2,99	0,76
p(value)^**^	0,003			0,137		0,014		0,524		0,055	
**Busca de informações**										
Com profissionais de saúde										
Não	248	2,59	0,59	2,62	0,51	2,65	0,44	2,96	0,57	2,71	0,45
Sim	302	2,77	0,56	2,66	0,47	2,72	0,42	2,95	0,62	2,71	0,43
p (value)^*^	0,001			0,331		0,079		0,891		0,954	

Nota: HLQ – Health Literacy Questionnaire; E1. Compreensão e apoio dos profissionais de saúde; E2. Informações suficientes para cuidar da saúde; E3. Cuidado ativo da saúde; E4. Suporte social para saúde; E5. Avaliação das informações em saúde; DM – Diabetes Mellitus; HAS – Hipertensão Arterial Sistêmica; SM – problema de saúde mental como depressão e ansiedade; UPA – Unidade de Pronto Atendimento 24h; APS – Atenção Primária à Saúde; DP – Desvio padrão amostral; *Teste t-Student para dois grupos independentes;

**Análise de Variância (One way); a e/ou b e/ou c Post Hoc Bonferroni, comparou as médias da variável estudada entre os grupos; se a médias foram marcadas com a letras sobrescritas iguais, elas não apresentam diferença estatisticamente significativa, mas apresentam significância se foram marcadas com a letras sobrescritas diferentes;

‡Outros – Doença Pulmonar Obstrutiva Crônica, asma, insuficiência renal, cardiopatias, acidente vascular cerebral, neoplasia, Parkinson, Alzheimer, doenças reumatológicas, obesidade;

†Outros – Consultório particular, CAPS. Um usuário referiu ter utilizado todos os serviços.

## DISCUSSÃO

Este estudo pioneiro no Brasil avaliou o LS de usuários de uma UPA 24h e identificou um perfil marcado por desigualdades sociodemográficas e clínicas, com implicações diretas para a comunicação, a tomada de decisão e a continuidade do cuidado em contextos de urgência. As maiores fortalezas situaram-se no suporte social para a saúde e na avaliação das informações, enquanto as principais fragilidades foram observadas na compreensão e apoio dos profissionais, no acesso a informações suficientes e no cuidado ativo da saúde. Esse padrão é concordante com achados internacionais que indicam menor LS em serviços de emergência em comparação a populações gerais, como observado na França e na Alemanha^(17,19)^.

A dimensão de suporte social apresentou as maiores médias, alinhando-se a evidências de que redes de apoio influenciam positivamente o bem-estar, a adesão terapêutica e a capacidade de lidar com situações agudas^(20)^. Da mesma forma, a boa avaliação das informações sugere que os usuários conseguem discriminar conteúdos confiáveis, achado também relatado entre adultos japoneses, que demonstraram habilidade de julgamento informacional relevante para o autocuidado^(21)^.

O LS variou significativamente conforme características sociodemográficas, reforçando sua associação com determinantes sociais da saúde. Mulheres apresentaram melhor desempenho na avaliação das informações, resultado apoiado por outro estudo realizado, que também sugere maior iniciativa feminina na busca e interpretação de dados de saúde^(22)^. No entanto, a literatura ressalta a necessidade de fortalecer o LS das mulheres, dada sua função central na gestão do cuidado familiar^(23)^.

Pessoas acima de 40 anos apresentaram escores mais altos em compreensão, apoio profissional, cuidado ativo e suporte social. Estudos internacionais apontam que essa vantagem pode decorrer da maior familiaridade acumulada com serviços de saúde e da experiência prévia na gestão de condições crônicas^(24)^.

A coabitação mostrou associação positiva com suporte social e habilidades de obtenção e avaliação de informações, reforçando a contribuição de redes afetivas e sociais, conforme apontado em estudos que destacam o papel do suporte emocional e instrumental no LS^(18,25)^. A renda familiar influenciou principalmente o suporte social e a avaliação da informação, achado consistente com pesquisas que identificam menor LS entre indivíduos com baixa renda, devido a desigualdades no acesso a recursos educativos, tecnologias e serviços^(26-27)^.

A escolaridade própria e a dos pais emergiram como determinantes centrais do LS, influenciando domínios relacionados à compreensão, informação suficiente e avaliação das informações. Evidências demonstram que a baixa escolaridade limita a compreensão de orientações e formulários, dificultando a tomada de decisões e a navegação no sistema de saúde^(28)^. A forte relação entre escolaridade parental e LS reforça a transmissão intergeracional de capital educacional e habilidades cognitivas relevantes para o cuidado.

O hábito de leitura foi um dos fatores comportamentais mais fortemente associados ao LS, corroborando estudos que mostram que a leitura regular favorece o desenvolvimento de habilidades cognitivas e interpretativas usadas na compreensão de orientações e na gestão da saúde^(29)^. Esse achado destaca oportunidades para intervenções educativas voltadas à promoção de práticas de leitura como estratégia de fortalecimento do LS.

Usuários com algum problema de saúde apresentaram maiores níveis de LS em comparação aos sem doenças, contrastando com dados franceses que mostraram maior LS limitado entre pacientes com condições crônicas^(19)^. Essa diferença pode ser explicada pelo maior contato desses usuários com o sistema de saúde, embora se reconheça que doenças como hipertensão e diabetes podem comprometer a cognição e influenciar negativamente o LS^(30)^.

A adesão ao tratamento esteve associada a melhores escores em várias dimensões do LS, em consonância com evidências que relacionam o LS adequado à compreensão de prescrições, ao autocuidado eficaz e à tomada de decisões seguras^(31)^. Usuários que procuraram serviços de saúde nos últimos 30 dias apresentaram melhor desempenho na obtenção de informações, sugerindo que o contato recente com profissionais pode reforçar orientações e promover maior clareza nas condutas^(32)^.

A busca de informações com profissionais também se associou à maior compreensão e apoio percebido, evidenciando o papel fundamental da comunicação profissional-usuário. Estratégias como o Teach-back, amplamente recomendadas, auxiliam na verificação da compreensão e reduzem falhas comunicacionais^(33)^. Considerando que o LS é concebido como tecnologia leve ancorada no vínculo, confiança e adaptação da linguagem ao contexto do usuário^(3)^, o profissional de enfermagem tem papel estratégico na redução das desigualdades informacionais.

Por fim, observa-se que a utilização frequente da UPA 24h pode refletir limitações no LS, expressas por dificuldades na adesão ao tratamento, na navegação pelos diferentes pontos da rede de atenção e na adequada interpretação das orientações em saúde, o que repercute em sobrecarga dos serviços e elevação dos custos assistenciais^(34)^. Nesse sentido, o fortalecimento do LS configura-se como estratégia fundamental para reduzir demandas desnecessárias, prevenir descompensações clínicas, subsidiar decisões informadas e promover maior equidade no acesso ao cuidado^(19)^. Ademais, torna-se imprescindível que os profissionais de saúde reconheçam a singularidade de cada usuário e incorporem estratégias de LS em sua prática assistencial, favorecendo o empoderamento dos indivíduos e ampliando sua capacidade de participar ativamente das decisões relacionadas à própria saúde e ao bem-estar^(35)^.

Esses achados reforçam o potencial do LS como ferramenta estratégica para qualificar a organização do cuidado na UPA, contribuindo para decisões mais seguras, comunicação efetiva e integração com a Rede de Atenção às Urgências.

Entre as limitações do estudo, destaca-se a exclusão de usuários classificados como risco vermelho e daqueles com déficit cognitivo, que, por razões éticas e clínicas, não puderam ser incluídos, o que pode restringir a compreensão do LS em grupos mais vulneráveis. Ademais, o delineamento transversal e a realização da pesquisa em uma única UPA de porte I limitam a generalização dos resultados. Recomenda-se que investigações futuras incorporem estratégias para a inclusão de perfis clínicos mais complexos, ampliem a representatividade dos participantes e considerem a realização de análises multivariadas, a fim de aprofundar a compreensão dos fatores associados ao LS nesse contexto.

## CONCLUSÃO

Este estudo demonstrou que o LS dos usuários de uma UPA 24h varia significativamente segundo fatores sociodemográficos e clínicos, com destaque para sexo, idade, estado civil, renda, escolaridade, hábito de leitura e adesão ao tratamento. As maiores fortalezas identificadas foram o suporte social e a avaliação das informações, enquanto persistiram fragilidades relacionadas à comunicação com profissionais, ao acesso a informações suficientes e ao manejo ativo da saúde. Os achados evidenciam a utilidade do HLQ para identificar padrões de LS nesse contexto assistencial, contribuindo para a compreensão das vulnerabilidades informacionais presentes nos serviços de urgência.

## Data Availability

Todo o conjunto de dados que dá suporte aos resultados deste estudo está disponível mediante solicitação ao autor correspondente.

# Translation: Alfabetización en salud y sus determinantes sociodemográficos y de salud

**DOI:** 10.1590/1980-220X-REEUSP-2025-0441es

**Authors:** Cristiane Ribas, Christiane de Fátima Colet, Edgar Noé Morelos-Garcia, Olga Maria Martins de Sousa Valentim, Márcio Strassburger, Fernanda Dal Maso Camera, Carolina Rodrigues Rusch, Adriane Cristina Bernat Kolankiewicz

**Affiliations:** 1 Universidade Regional do Noroeste do Estado do Rio Grande do Sul, Programa de Pós-Graduação Strictu Sensu em Atenção Integral à Saúde, Ijuí, RS, Brasil.; 2 Universidade Autônoma de Tamaulipas, Tamaulipas, Mexico.; 3 Universidade de Lisboa, Escola Superior de Enfermagem de Lisboa, Centro de Investigação, Inovação e Desenvolvimento em Enfermagem de Lisboa, Lisboa, Portugal.; 4 Universidade do Porto, Escola Superior de Enfermagem, RISE-Health, Porto, Portugal.; 5 Universidade Regional do Noroeste do Estado do Rio Grande do Sul, Curso de Fisioterapia, Ijuí, RS, Brasil.; 6 Universidade Regional Integrada do Alto Uruguai e das Missões, Programa de Pós-Graduação Strictu Sensu em Atenção Integral à Saúde, Erechim, RS, Brasil.; 7 Universidade Regional do Noroeste do Estado do Rio Grande do Sul, Curso de Enfermagem, Ijuí, RS, Brasil.

**Keywords:** Servicios Médicos de Urgencia, Alfabetización en Salud, Conductas Relacionadas con la Salud; Características de la Población.

## Abstract

**Objetivo:**

Evaluar la alfabetización en salud y sus asociaciones sociodemográficas y clínicas entre los usuarios de un servicio de urgencias.

**Método:**

Estudio transversal realizado en un servicio de urgencias de 24 horas en el sur de Brasil, con una muestra de 550 usuarios (≥18 años). Se aplicó un cuestionario sociodemográfico y clínico, así como las escalas del 1 al 5 del Health Literacy Questionnaire. Se realizaron análisis descriptivos e inferenciales (prueba t de Student y ANOVA; p ≤ 0,05).

**Resultados:**

Las medias más altas se registraron en “apoyo social” (2,95 ± 0,60) y “evaluación de la información” (2,71 ± 0,44), y las más bajas en “información suficiente” (2,64 ± 0,49) y “comprensión profesional” (2,69 ± 0,58). Las puntuaciones más altas se asociaron con el sexo femenino (p = 0,021), edad >40 años (p ≤ 0,044), vivir en unión libre (p = 0,045; p = 0,010), convivencia (p = 0,008), mayor ingreso (p = 0,006; p = 0,007) y mayor nivel educativo propio y de los padres (p ≤ 0,038). El hábito de lectura se asoció con todas las dimensiones (p ≤ 0,005). Los problemas de salud (p ≤ 0,023), la adherencia al tratamiento (p < 0,05), la búsqueda reciente de servicios (p ≤ 0,037) y la búsqueda de información con profesionales (p = 0,001) también influyeron en las puntuaciones.

**Conclusión:**

La alfabetización en salud varió significativamente según las características sociodemográficas y clínicas entre los usuarios.

## INTRODUCCIÓN

El Sistema Único de Salud (SUS) se estructura en tres niveles jerárquicos, según los niveles de atención sanitaria: Atención Primaria, complejidad media y alta complejidad. La articulación de estos niveles es esencial para una atención adecuada y eficiente, con el objetivo de brindar una atención integral al paciente^(1)^. Como parte de los servicios de salud del SUS, la Unidad de Atención de Urgencias 24 horas (UPA 24h) es un servicio de complejidad intermedia entre la Atención Primaria de Salud (APS) y la Red Hospitalaria. El servicio tiene como objetivo garantizar una atención eficaz a los usuarios afectados por cuadros agudos de naturaleza clínica y en situaciones quirúrgicas y de trauma^(2)^.

En este contexto, la Alfabetización en Salud (AS) es esencial para que la población pueda cuidar su salud, realizar el seguimiento, adherirse al tratamiento y tomar decisiones en pro de su salud, lo que puede impactar directamente en la calidad de la salud de los individuos y de la comunidad^(3)^. En 2020, el *U. S. Department of Health and Human Services*(Departamento de Salud y Servicios Humanos de los Estados Unidos) definió la AS – *Health Literacy* como “el grado de capacidad que tiene cada individuo para encontrar, comprender y utilizar información y servicios para tomar decisiones y medidas en favor de su propia salud y la de otros”^(4)^.

De manera convergente, la Organización Mundial de la Salud amplió la definición al afirmar que la AS no es solo un atributo individual, sino también un producto de las condiciones sociales, económicas e institucionales. Por lo tanto, corresponde a los servicios de salud crear entornos, materiales y prácticas comunicacionales que apoyen la comprensión, la toma de decisiones y el ejercicio de la autonomía, destacando el papel de las organizaciones de salud en el desarrollo de las habilidades de AS de la población^(5)^.

La AS tiene múltiples conceptos y está relacionada e intercalada entre los campos de la salud y la educación, con el objetivo de mejorar la calidad de vida y la autonomía del individuo. Para evaluar las potencialidades y debilidades de la AS de los individuos^(6)^, una de las herramientas utilizadas es el *Health Literacy Questionnaire* (HLQ)^(7)^. Se trata de un instrumento multidimensional, aplicable en diferentes culturas y países con el fin de evaluar la AS, más allá de las habilidades cognitivas de lectura y aritmética^(7–8)^.

Se han realizado estudios sobre la AS en diferentes contextos asistenciales y poblacionales, incluyendo usuarios de la APS con afecciones crónicas, como hipertensión arterial y diabetes mellitus tipo II^(9)^, así como personas sometidas a terapia dialítica^(10)^, personas en la sala de emergencias^(11)^, con enfermedad coronaria^(12)^, pacientes con cáncer hematológico^(13)^, profesionales de la salud^(14)^, cuidadores domiciliarios^(15)^ y personas que viven con trastornos mentales graves y persistentes^(16)^. En general, estas investigaciones evidencian asociaciones consistentes entre la AS, los factores sociodemográficos, la adherencia terapéutica y la capacidad de autogestión del cuidado. Sin embargo, dichos estudios se centran predominantemente en escenarios electivos o de seguimiento longitudinal, por lo que la producción científica orientada a los servicios de urgencias y emergencias sigue siendo limitada.

En el ámbito de los servicios de emergencia, la literatura incluye investigaciones realizadas con cuidadores en servicios de urgencias pediátricas^(17)^, análisis comparativos con la población general^(18)^ y estudios que abordan factores relacionados con la autogestión en contextos hospitalarios de emergencia^(19)^.

Sin embargo, se observa una escasez de evidencia dirigida a los servicios de urgencias intermedios, como las UPA 24h, que ocupan una posición estratégica en la Red de Atención a las Urgencias y se caracterizan por una elevada demanda asistencial, vulnerabilidad comunicacional y la necesidad de decisiones rápidas. En este escenario, las limitaciones en el nivel de AS pueden comprometer la comprensión de las orientaciones sanitarias, la adherencia al tratamiento y el uso adecuado de los diferentes puntos de la red asistencial. Por lo tanto, analizar el nivel de AS de los usuarios de las UPA 24h y su relación con las características sociodemográficas y clínicas contribuye a reducir la brecha de conocimiento y a mejorar la práctica asistencial. Este estudio parte de la hipótesis de que los usuarios de la UPA 24h presentan niveles de AS asociados a características sociodemográficas, educativas, conductuales y clínicas, las cuales se relacionan con la comprensión de las orientaciones, la adherencia al tratamiento y el patrón de utilización de los servicios de salud.

En este contexto, este estudio tiene como objetivo evaluar la alfabetización en salud y sus asociaciones sociodemográficas y clínicas entre los usuarios de la unidad de atención de urgencias.

## MÉTODO

### Tipo y Lugar del Estudio

Estudio transversal, conforme a las *guidelines* STROBE, realizado en una Unidad de Atención de Urgencias (UPA) 24h de nivel I en Rio Grande do Sul, Brasil, que atiende un promedio de 5.651 casos al mes. La UPA cuenta con infraestructura para la atención de adultos y niños, funciona las 24 horas, los siete días de la semana, con un equipo mínimo compuesto por médicos, enfermeros y técnicos de enfermería, además de apoyo de laboratorio y radiología.

### Definición de la Muestra

La muestra incluyó a usuarios ≥18 años atendidos entre enero y febrero de 2024. Se excluyó a las personas con déficit cognitivo identificado por desorientación, incoherencia o incapacidad para comprender información, así como a aquellas que no podían comunicarse verbalmente o responder al instrumento. El déficit cognitivo fue evaluado por un enfermero capacitado, antes de la recolección de datos. Se excluyó a los pacientes clasificados como riesgo rojo, por tratarse de casos que requieren atención inmediata, caracterizados por inestabilidad clínica grave, riesgo inminente de muerte y necesidad de intervención rápida.

El cálculo de la muestra consideró una población de 5.651 usuarios, un error del 4% y un nivel de confianza del 95%, lo que dio como resultado 543 participantes. El muestreo fue probabilístico, seleccionándose 1 de cada 10 usuarios elegibles, tras el sorteo del punto inicial. Se utilizó la fórmula:


$${\text{n = [N · Z}}^{\text{2}}{\text{· p (1-p)]/[Z}}^{\text{2}}{\text{· p (1-p) + e}}^{\text{2}}\text{(N-1)]}$$

### Recolección de Datos

Se invitó a los participantes a participar en el estudio desde su ingreso a la UPA. El cuestionario sociodemográfico incluyó edad, sexo, estado civil, convivencia, ingreso familiar, nivel educativo del participante y nivel educativo de los padres (sin instrucción/no sabe, educación primaria, educación secundaria o educación superior/posgrado).

También se recopilaron variables de comportamiento y de estado de salud: hábito de lectura, evaluado mediante una pregunta dicotómica (“¿Tiene el hábito de leer?”), siendo “sí” cuando existía una lectura rutinaria de materiales impresos o digitales; y la adherencia al tratamiento, investigada mediante autoinforme (“¿Sigue regularmente las orientaciones y los tratamientos prescritos?”), considerándose “sí” cuando el individuo cumplía la mayor parte de las recomendaciones. También se registraron la presencia y el tipo de problema de salud, la búsqueda de servicios en los últimos 30 días y la fuente de búsqueda de información. La recolección se realizó presencialmente a través de *Google Forms*. El HLQ, validado en Brasil ^(7–8)^, evaluó la AS mediante las escalas 1 a 5, de acuerdo con las recomendaciones originales^(8)^. Su uso se definió mediante la realización de una evaluación multidimensional. Aunque el HLQ contempla nueve escalas que abarcan dimensiones individuales, interpersonales y organizacionales de la AS, este estudio priorizó las escalas del 1 al 5 por representar competencias directamente movilizadas en el momento de la atención en la UPA, como la comprensión de las orientaciones profesionales, la evaluación de la información recibida y el compromiso con el cuidado.

### Análisis y Tratamiento de los Datos

El análisis estadístico se realizó en IBM SPSS 25.0, adoptando un nivel de significación del 5%. Se llevaron a cabo análisis descriptivos, la prueba de normalidad de Kolmogorov–Smirnov y comparaciones entre grupos mediante la t de Student o ANOVA unidireccional con post-hoc de Bonferroni. Además, se compararon las escalas del HLQ según el nivel educativo, el nivel educativo de los padres, el hábito de lectura, la presencia y el tipo de problema de salud, la búsqueda de servicios de salud en los últimos 30 días, la fuente de búsqueda de información y la adherencia al tratamiento, empleando las mismas pruebas según correspondiera. En el análisis comparativo, los ingresos familiares se clasificaron en tres rangos: hasta R$ 2.900,00; por encima de R$ 2.900,00 hasta R$ 4.900,00; y por encima de R$ 4.900,00.

### Aspectos Éticos

El uso del HLQ fue autorizado por los titulares de los derechos de autor (Swinburne University) (hlinfo@swin.edu.au) y el proyecto fue aprobado por el Comité de Ética en Investigación (CAAE n.º 75589123800005350, Dictamen n.º 6570946, 11 de diciembre de 2023). La recolección de datos se realizó mediante el consentimiento informado de los participantes en el Término de Consentimiento Libre e Informado (TCLE).

## RESULTADOS

Participaron 550 usuarios de la UPA 24h, en su mayoría mujeres (54,5%) y adultos de entre 18 y 40 años (54,5%). Predominaron las personas de raza blanca (71,6%), con educación secundaria (52,3%) y que convivían con otras personas (86,9%).

La distribución según el estado civil se mostró equilibrada, mientras que los ingresos mensuales presentaron una distribución relativamente homogénea entre los tres rangos de ingresos analizados (Tabla 1).

**Tabla 1:** Caracterización sociodemográfica y de las condiciones de salud de los usuarios que acuden a la UPA 24h – Ijuí, RS, Brasil, 2024.

Variables	Categorías	n	%
Sexo	Mujer	300	54,5%
	Hombre	250	45,5%
Edad	De 18 a 40 años	300	54,5%
	Más de 40 años	250	45,5%
Raza	Blanca	394	71,6%
	No blanca^*^	156	28,4%
Estado civil	Vive en pareja	272	49,4%
	No vive en pareja	278	50,6%
Cohabitación	Vive con alguien	478	86,9%
	Vive solo	72	13,1%
Escolaridad	Hasta secundaria	469	85,3%
	Universitaria/posgrado	81	14,7%
Ingreso familiar (R$)	Hasta 2.900,00	185	33,6%
	Entre 2.900,00 y 4.900,00	180	32,7%
	Más de 4.900,00	180	32,7%

Nota: *No blancos: aquellos que se identifican como asiáticos, pardos y negros.

El análisis del estado de salud de los pacientes reveló que la mitad de los usuarios no informó de ninguna enfermedad crónica (303; 55,1%) y el resto (247; 44,9%) informaron que padecían diversas patologías, como diabetes mellitus (9; 1,6%), hipertensión arterial sistémica (HAS) (81; 14,7%), diabetes mellitus y HAS concomitantes (24; 4,3%), trastorno mental (56; 10,1%), entre otras condiciones de salud. La evaluación de la AS identificó como puntos fuertes el “apoyo social para la salud” y la “evaluación de la información en salud” y como debilidades las demás escalas (Tabla 2), considerando las medias más altas observadas, aunque con diferencias discretas entre las escalas.

**Tabla 2:** Promedios de alfabetización en salud de los usuarios que acuden a la UPA 24h en relación con las escalas 01 a 05 del Cuestionario de Alfabetización en Salud (Health Literacy Questionnaire) – Ijuí, RS, Brasil, 2024.

Escalas	Media	Desvío estándar
1. Comprensión y apoyo por parte de los profesionales de la salud	2,69	0,58
2. Información suficiente para cuidar la salud	2,64	0,49
3. Cuidado activo de la salud	2,69	0,43
4. Apoyo social para la salud	2,95	0,60
5. Evaluación de la información en salud	2,71	0,44

La AS varió según las características sociodemográficas. Se observó una variación significativa en la AS según el sexo, la edad, el estado civil, la convivencia y los ingresos. Las mujeres presentaron medias más altas en el dominio de la evaluación de la información (p = 0,021). Los usuarios mayores de 40 años mostraron niveles superiores en comprensión, cuidado activo y apoyo social (p < 0,05). Vivir en pareja se asoció con puntuaciones más altas en información y evaluación de la información. La convivencia influyó en el apoyo social (p = 0,008). Los ingresos más altos mostraron un mejor desempeño en apoyo social y evaluación de la información (p < 0,01), lo que indica una influencia socioeconómica en el alfabetización en salud. (Tabla 3).

**Tabla 3:** Promedios de alfabetización en salud de los usuarios que acuden a la UPA 24h, según las características sociodemográficas – Ijuí, RS, Brasil, 2024.

Características socio demográficas	HLQ										
	n	E1		E2		E3		E4		E5	
		Media	DE	Media	DE	Media	DE	Media	DE	Media	DE
**Sexo**										
Hombres	250	2,64	0,59	2,60	0,47	2,68	0,44	2,66	0,61	2,67	0,47
Mujeres	300	2,73	0,57	2,66	0,48	2,75	0,42	2,75	0,63	2,75	0,41
p (valor)^*^	0,061			0,122		0,837		0,169		0,021	
**Rango de edad**										
De 18 a 40 años	300	2,57	0,57	2,60	0,48	2,64	0,42	2,91	0,60	2,70	0,41
Más de 40 años	250	2,84	0,56	2,67	0,50	2,75	0,44	3,01	0,57	2,71	0,48
p (valor)^*^	< 0,001			0,175		0,002		0,044		0,129	
**Estado civil**										
Vive en pareja	272	2,72	0,57	2,68	0,48	2,72	0,42	3,00	0,60	2,76	0,44
No vive en pareja	278	2,66	0,59	2,60	0,49	2,65	0,44	2,91	0,59	2,67	0,43
p (valor)^*^	0,186			0,045		0,051		0,082		0,010	
**Convivencia**										
Vive solo	72	2,75	0,56	2,64	0,53	2,67	0,42	2,78	0,65	2,70	0,45
Vive con alguien	478	2,68	0,58	2,64	0,48	2,69	0,43	2,98	0,59	2,72	0,44
p (valor)^*^	0,340			0,999		0,719		0,008		0,844	
**Ingresos**										
Hasta 2.900,00 R$	185	2,67	0,61	2,60	0,49	2,64	0,43	2,87b	0,66	2,64b	0,45
De más de 2.900,00 R$ a 4.900,00 R$	180	2,68	0,53	2,62	0,47	2,69	0,42	2,93a,b	0,58	2,71a,b	0,39
De más de 4.900,00 R$	185	2,72	0,60	2,70	0,50	2,73	0,44	3,06^a^	0,54	2,79^a^	0,47
p (valor)^*^	0,677			0,114		0,185		0,006		0,007	

Nota: HLQ – Cuestionario de Alfabetización en Salud; E1. Comprensión y apoyo de los profesionales de la salud; E2. Información suficiente para cuidar la salud; E3. Cuidado activo de la salud; E4. Apoyo social para la salud; E5. Evaluación de la información en salud; DE – Desviación estándar muestral. *Prueba t de Student para grupos independientes; **Análisis de varianza (unidireccional).

La Tabla 4 muestra que los niveles de AS fueron significativamente mayores en los usuarios con educación superior o posgrado, especialmente en la comprensión y el apoyo de los profesionales, el acceso a información suficiente, el cuidado activo y la evaluación de la información (p < 0,05). El nivel educativo de la madre y del padre también se relacionó positivamente con la AS, destacando la influencia de los padres en la comprensión y la evaluación de la información de salud. No se observaron diferencias significativas en el apoyo social según el nivel educativo.

**Tabla 4:** Promedios de conocimientos en salud de los usuarios que acuden a la UPA 24h, según su nivel de estudios y el de sus padres – Ijuí, RS, Brasil, 2024.

Niveles de escolaridad	HLQ										
	n	E1		E2		E3		E4		E5	
		Media	DE	Media	DE	Media	DE	Media	DE	Media	DE
**Nivel educativo**										
Educación primaria	181	2,73^a,b^	0,55	2,56^b^	0,45	2,66^b^	0,42	2,59	0,44	2,63^b^	0,44
Educación secundaria	288	2,62^b^	0,60	2,62^b^	0,51	2,67^b^	0,43	2,69	0,44	2,73^b^	0,44
Educación superior o posgrado	81	2,83^a^	0,56	2,87^a^	0,43	2,80^a^	0,47	2,84	0,41	2,84^a^	0,41
p(valor)^*^	0,010			< 0,001		0,042		0,126		0,001	
**Nivel educativo de la madre**										
Sin estudios o no sabe	156	2,79^a^	0,55	2,64	0,49	2,71	0,47	3,02	0,54	2,60^c^	0,46
Educación primaria	281	2,62^b^	0,61	2,63	0,50	2,69	0,43	2,95	0,64	2,74^b^	0,46
Educación secundaria	79	2,68^b^	0,51	2,64	0,45	2,63	0,37	2,91	0,49	2,76^b^	0,33
Educación superior o posgrado	34	2,77^a^	0,62	2,76	0,48	2,72	0,42	2,79	0,65	2,89^a^	0,25
p(valor)^*^	0,023			0,541		0,603		0,198		0,001	
**Nivel educativo del padre**										
Sin estudios o no sabe	191	2,77^a^	0,60	2,61	0,51	2,66	0,46	3,01	0,55	2,62^b^	0,48
Educación primaria	265	2,62^b^	0,58	2,64	0,48	2,70	0,42	2,88	0,67	2,74^a,b^	0,43
Educación secundaria	67	2,71^a,b^	0,51	2,68	0,50	2,70	0,38	3,06	0,41	2,81ª	0,38
Educación superior o posgrado	27	2,79^a^	0,56	2,76	0,38	2,73	0,47	3,01	0,47	2,82ª	0,27
p(valor)^*^	0,038			0,395		0,837		0,060		0,003	

Nota: HLQ – Cuestionario de Alfabetización en Salud; E1. Comprensión y apoyo de los profesionales de la salud; E2. Información suficiente para cuidar la salud; E3. Cuidado activo de la salud; E4. Apoyo social para la salud; E5. Evaluación de la información en salud; DE – Desviación estándar muestral; *Análisis de varianza (unidireccional); a y/o b Post Hoc Bonferroni: comparó las medias de la variable estudiada entre los grupos; si las medias se marcaron con las mismas letras superpuestas, no presentan una diferencia estadísticamente significativa, pero sí la presentan si se marcaron con letras superpuestas diferentes.

La Tabla 5 muestra que los usuarios con hábito de lectura presentaron mejores puntuaciones en todas las dimensiones del HLQ (p < 0,005), lo que indica una mayor AS. Los problemas de salud se asociaron con puntuaciones más altas en E1 y E2 (p < 0,05), y aquellos con múltiples afecciones mostraron mayor apoyo social (E4) (p < 0,001). La adherencia al tratamiento se relacionó con mejores resultados en casi todas las dimensiones (p < 0,05). Los usuarios que buscaron atención en los últimos 30 días obtuvieron puntuaciones más altas en E1 y E2 (p < 0,05), destacando el grupo “otros” en E1 y E3. La búsqueda de información con profesionales elevó las puntuaciones en E1 (p = 0,001). Estos hallazgos refuerzan la importancia del hábito de lectura y de la adherencia al tratamiento para la AS en la UPA 24h.

**Tabla 5:** Promedios de alfabetización en salud de los usuarios que acuden a la UPA 24h, según sus hábitos y su estado de salud – Ijuí, RS, Brasil, 2024.

Condiciones de salud	HLQ										
	n	E1		E2		E3		E4		E5	
		Media	DE	Media	DE	Media	DE	Media	DE	Media	DE
**Hábito de lectura**										
Si	253	2,82	0,55	2,72	0,46	2,75	0,41	3,03	0,56	2,80	0,42
No	297	2,58	0,58	2,57	0,50	2,63	0,45	2,89	0,62	2,64	0,45
p (valor) ^*^	< 0,001			< 0,001		0,001		0,005		< 0,001	
**Problema de Salud**										
Si	247	2,82	0,56	2,69	0,50	2,72	0,44	2,99	0,65	2,74	0,44
No	303	2,58	0,58	2,60	0,48	2,66	0,42	2,93	0,55	2,69	0,44
p (valor) ^*^	< 0,001			0,023		0,130		0,239		0,139	
**Cuáles problemas de salud**										
DM	9	2,78	0,36	2,50^c^	0,35	2,73	0,32	3,13^b^	0,85	2,51	0,40
HAS	81	2,79	0,43	2,62bc	0,42	2,71	0,27	2,93^bb^	0,58	2,74	0,36
DM+HAS	24	2,92	0,25	2,68^b^	0,34	2,79	0,24	2,98^a,b^	0,47	2,56	0,33
SM	56	2,70	0,61	2,62^b^	0,48	2,63	0,42	2,73^c^	0,74	2,80	0,34
Otros^‡^	77	2,92	0,69	2,85^a^	0,61	2,76	0,62	3,22^a^	0,62	2,80	0,59
p(valor)^**^	0,186			0,018		0,491		< 0,001		0,066	
**Adherencia al tratamiento de Salud**										
Si	215	2,85	0,57	2,72	0,49	2,75	0,45	3,02	0,63	2,98	0,77
No o a veces	335	2,63	0,43	2,54	0,53	2,55	0,36	2,77	0,76	2,85	0,62
p (valor)^*^	0,023			0,046		0,009		0,027		0,106	
**¿Ha acudido a algún centro de salud en los últimos 30 días?**										
Si	258	2,74	0,57	2,70	0,49	2,69	0,44	2,96	0,64	2,73	0,45
No	292	2,64	0,59	2,59	0,49	2,68	0,43	2,95	0,56	2,70	0,43
p (valor)^*^	0,037			0,01		0,863		0,868		0,309	
**A cuál servicio acudió**										
UPA	96	2,64^b^	0,48	2,68	0,43	2,59^b^	0,38	2,89	0,64	2,95	0,64
APS	138	2,77^a,b^	0,61	2,68	0,52	2,71^a,b^	0,42	2,96	0,65	2,73	0,35
Otros^†^	19	3,29^a^	0,43	2,74	0,66	3,06^a^	0,61	3,19	0,74	2,99	0,76
p(valor)^**^	0,003			0,137		0,014		0,524		0,055	
**Busca de informaciones**										
Con profesionales de salud										
No	248	2,59	0,59	2,62	0,51	2,65	0,44	2,96	0,57	2,71	0,45
Si	302	2,77	0,56	2,66	0,47	2,72	0,42	2,95	0,62	2,71	0,43
p (valor)^*^	0,001			0,331		0,079		0,891		0,954	

Nota: HLQ – Cuestionario de alfabetización en salud; E1. Comprensión y apoyo de los profesionales de la salud; E2. Información suficiente para cuidar la salud; E3. Cuidado activo de la salud; E4. Apoyo social para la salud; E5. Evaluación de la información en salud; DM – Diabetes Mellitus; HAS – Hipertensión Arterial Sistémica; SM – Problemas de salud mental como depresión y ansiedad; UPA – Unidad de Atención de Urgencias 24h; APS – Atención Primaria de Salud; DE – Desviación estándar muestral; *Prueba t de Student para dos grupos independientes;

**Análisis de varianza (unidireccional); a y/o b y/o c Post Hoc Bonferroni, comparó las medias de la variable estudiada entre los grupos; si las medias se marcaron con letras superpuestas iguales, no presentan una diferencia estadísticamente significativa, pero sí la presentan si se marcaron con letras superpuestas diferentes;

‡Otros: enfermedad pulmonar obstructiva crónica, asma, insuficiencia renal, cardiopatías, accidente cerebrovascular, neoplasia, Parkinson, Alzheimer, enfermedades reumatológicas, obesidad;

†Otros: consultorio privado, CAPS. Un usuario mencionó haber utilizado todos los servicios.

## DISCUSIÓN

Este estudio pionero en Brasil evaluó la AS de los usuarios de una UPA 24h e identificó un perfil marcado por desigualdades sociodemográficas y clínicas, con implicaciones directas para la comunicación, la toma de decisiones y la continuidad de la atención en contextos de urgencia. Las mayores fortalezas se situaron en el apoyo social para la salud y en la evaluación de la información, mientras que las principales debilidades se observaron en la comprensión y el apoyo de los profesionales, en el acceso a información suficiente y en el cuidado activo de la salud. Este patrón concuerda con hallazgos internacionales que indican una menor AS en los servicios de emergencia en comparación con la población general, como se ha observado en Francia y Alemania^(17,19)^.

La dimensión del apoyo social presentó los promedios más altos, en consonancia con las evidencias de que las redes de apoyo influyen positivamente en el bienestar, la adherencia terapéutica y la capacidad para lidiar con situaciones agudas^(20)^. Del mismo modo, la buena evaluación de la información sugiere que los usuarios logran distinguir contenidos confiables, hallazgo también reportado entre adultos japoneses, quienes demostraron una capacidad de juicio informativo relevante para el autocuidado^(21)^.

La AS varió significativamente según las características sociodemográficas, lo que refuerza su asociación con los determinantes sociales de la salud. Las mujeres obtuvieron mejores resultados en la evaluación de la información, un resultado respaldado por otro estudio realizado, que también sugiere una mayor iniciativa femenina en la búsqueda e interpretación de datos de salud^(22)^. Sin embargo, la literatura destaca la necesidad de fortalecer la AS de las mujeres, dada su función central en la gestión del cuidado familiar^(23)^.

Las personas mayores de 40 años obtuvieron puntuaciones más altas en comprensión, apoyo profesional, cuidado activo y apoyo social. Estudios internacionales indican que esta ventaja puede deberse a una mayor familiaridad acumulada con los servicios de salud y a la experiencia previa en el manejo de afecciones crónicas^(24)^.

La convivencia mostró una asociación positiva con el apoyo social y las habilidades para obtener y evaluar información, lo que refuerza la contribución de las redes afectivas y sociales, tal como se señala en estudios que destacan el papel del apoyo emocional e instrumental en la AS^(18,25)^. Los ingresos familiares influyeron principalmente en el apoyo social y la evaluación de la información, un hallazgo consistente con investigaciones que identifican una menor AS entre individuos de bajos ingresos, debido a las desigualdades en el acceso a recursos educativos, tecnologías y servicios^(26-27)^.

El nivel educativo propio y el de los padres surgieron como determinantes centrales de la AS, influyendo en dominios relacionados con la comprensión, la información suficiente y la evaluación de la información. Las evidencias demuestran que el bajo nivel educativo limita la comprensión de las orientaciones y los formularios, lo que dificulta la toma de decisiones y la navegación por el sistema de salud^(28)^. La fuerte relación entre el nivel educativo de los padres y la AS refuerza la transmisión intergeneracional del capital educativo y las habilidades cognitivas relevantes para el cuidado.

El hábito de lectura fue uno de los factores conductuales más fuertemente asociados a la AS, lo que corrobora estudios que muestran que la lectura regular favorece el desarrollo de habilidades cognitivas e interpretativas utilizadas en la comprensión de las orientaciones y en la gestión de la salud^(29)^. Este hallazgo destaca oportunidades para intervenciones educativas orientadas a la promoción de prácticas de lectura como estrategia para fortalecer la AS.

Los usuarios con algún problema de salud presentaron mayores niveles de AS en comparación con los que no padecían enfermedades, lo que contrasta con datos franceses que mostraron una AS más limitado entre pacientes con afecciones crónicas^(19)^. Esta diferencia puede explicarse por el mayor contacto de estos usuarios con el sistema de salud, aunque se reconoce que enfermedades como la hipertensión y la diabetes pueden comprometer la cognición e influir negativamente en la AS^(30)^.

La adherencia al tratamiento se asoció con mejores puntuaciones en varias dimensiones de la AS, en consonancia con las evidencias que relacionan una AS adecuada con la comprensión de las recetas, el autocuidado eficaz y la toma de decisiones seguras^(31)^. Los usuarios que buscaron servicios de salud en los últimos 30 días presentaron un mejor desempeño en la obtención de información, lo que sugiere que el contacto reciente con profesionales puede reforzar las orientaciones y promover una mayor claridad en las conductas^(32)^.

La búsqueda de información con profesionales también se asoció con una mayor comprensión y apoyo percibido, lo que evidencia el papel fundamental de la comunicación entre el profesional y el usuario. Estrategias como el “Teach-back”, ampliamente recomendadas, ayudan a verificar la comprensión y reducen las fallas comunicacionales^(33)^. Considerando que la AS se concibe como una tecnología ligera basada en el vínculo, la confianza y la adaptación del lenguaje al contexto del usuario^(3)^, el profesional de enfermería tiene un papel estratégico en la reducción de las desigualdades informacionales.

Por último, se observa que el uso frecuente de la UPA 24h puede reflejar limitaciones en la AS, expresadas por dificultades en la adherencia al tratamiento, en la navegación por los diferentes puntos de la red de atención y en la interpretación adecuada de las orientaciones en salud, lo que repercute en la sobrecarga de los servicios y el aumento de los costos asistenciales^(34)^. En este sentido, el fortalecimiento de la AS se configura como una estrategia fundamental para reducir demandas innecesarias, prevenir descompensaciones clínicas, respaldar decisiones informadas y promover una mayor equidad en el acceso a la atención^(19)^. Además, resulta imprescindible que los profesionales de la salud reconozcan la singularidad de cada usuario e incorporen estrategias de AS en su práctica asistencial, favoreciendo el empoderamiento de las personas y ampliando su capacidad para participar activamente en las decisiones relacionadas con su propia salud y bienestar^(35)^.

Estos hallazgos refuerzan el potencial de la AS como herramienta estratégica para mejorar la organización de la atención en la UPA, contribuyendo a decisiones más seguras, una comunicación efectiva y la integración con la Red de Atención a las Urgencias.

Entre las limitaciones del estudio, destaca la exclusión de usuarios clasificados como riesgo rojo y de aquellos con déficit cognitivo, que, por razones éticas y clínicas, no pudieron ser incluidos, lo que puede restringir la comprensión de la AS en los grupos más vulnerables. Además, el diseño transversal y la realización de la investigación en una sola UPA de tamaño I limitan la generalización de los resultados. Se recomienda que futuras investigaciones incorporen estrategias para la inclusión de perfiles clínicos más complejos, amplíen la representatividad de los participantes y consideren la realización de análisis multivariados, con el fin de profundizar en la comprensión de los factores asociados a la AS en este contexto.

## CONCLUSIÓN

Este estudio demostró que la AS de los usuarios de una UPA 24 horas varía significativamente según factores sociodemográficos y clínicos, destacando el sexo, la edad, el estado civil, los ingresos, el nivel educativo, el hábito de lectura y la adherencia al tratamiento. Las mayores fortalezas identificadas fueron el apoyo social y la evaluación de la información, mientras que persistieron debilidades relacionadas con la comunicación con los profesionales, el acceso a información suficiente y el manejo activo de la salud. Los hallazgos evidencian la utilidad del HLQ para identificar patrones de AS en este contexto asistencial, contribuyendo a la comprensión de las vulnerabilidades informacionales presentes en los servicios de urgencias.

## Data Availability

El conjunto de datos que respaldan las conclusiones de este estudio está disponible previa solicitud al autor correspondiente.
